# Causal interaction following the alteration of target region activation during motor imagery training using real-time fMRI

**DOI:** 10.3389/fnhum.2013.00866

**Published:** 2013-12-16

**Authors:** Xiaojie Zhao, Hang Zhang, Sutao Song, Qing Ye, Jia Guo, Li Yao

**Affiliations:** ^1^College of Information Science and Technology, Beijing Normal UniversityBeijing, China; ^2^Paul C. Lauterbur Research Centers for Biomedical Imaging, Shenzhen Institutes of Advanced Technology, Chinese Academy of SciencesShenzhen, China; ^3^School of Education and Psychology, Jinan UniversityJinan, China; ^4^State Key Laboratory of Cognitive Neuroscience and Learning, Beijing Normal UniversityBeijing, China

**Keywords:** real-time fMRI, motor imagery, Granger causality, interaction, training

## Abstract

Motor imagery training is an effective approach for motor skill learning and motor function rehabilitation. As a novel method of motor imagery training, real-time fMRI (rtfMRI) enables individuals to acquire self-control of localized brain activation, achieving desired changes in behavior. The regulation of target region activation by rtfMRI often alters the activation of related brain regions. However, the interaction between the target region and these related regions is unclear. The Granger causality model (GCM) is a data-driven method that can explore the causal interaction between brain regions. In this study, we employed rtfMRI to train subjects to regulate the activation of the ipsilateral dorsal premotor area (dPMA) during motor imagery training, and we calculated the causal interaction of the dPMA with other motor-related regions based on the GCM. The results demonstrated that as the activity of the dPMA changed during rtfMRI training, the interaction of the target region with other related regions became significantly altered, and behavioral performance was improved after training. The altered interaction primarily exhibited as an increased unidirectional interaction from the dPMA to the other regions. These findings support the dominant role of the dPMA in motor skill learning via rtfMRI training and may indicate how activation of the target region interacts with the activation of other related regions.

## Introduction

The real-time functional MRI (rtfMRI) technique is a novel training method that enables the monitoring of changes in brain activation and trains subjects to voluntarily control the activation of target regions by feedback in order to induce the associated behavioral alteration. Evidence from several studies has demonstrated that activation of some motor cortical areas can be regulated through rtfMRI training, such as the sensorimotor cortex (deCharms et al., 2004), the primary motor cortex (Yoo et al., 2008), and the ventral premotor area (vPMA) (Sitaram et al., 2011). These studies also demonstrated that regulating on the target region could lead to the alteration of activation in the other motor-related regions by rtfMRI training. However, the interaction of the target region with other regions, which facilitates exploring the neural mechanism underlying the rtfMRI training, is unclear.

The Granger causality model (GCM), introduced by Granger in 1969, is an important tool for exploring the dynamic causal interactions between two time series (Granger, 1969). It was first applied to electroencephalography and magneto-encephalography data (Kamiñski et al., 2001; Brovelli et al., 2004) and subsequently to fMRI data (Goebel et al., 2003; Roebroeck et al., 2005; Gao et al., 2008). The causal relationships calculated from brain data can help us understand how different brain regions coordinate and interact directionally. Compared with other methods for exploring the causal relationships used in brain data, such as structural equation modeling (SEM) (McLntosh and Gonzalez-Lima, 1994) and dynamic causal modeling (DCM) (Friston et al., 2003), GCM is not the hypothesis-driven method but data-driven method, which is of benefit to describe the directional interaction among brain regions from fMRI data itself.

Motor imagery is considered to be an effective strategy for motor skill learning and motor function, especially for complete loss of motion (Sharma et al., 2006). Neuroimaging studies of motor imagery training found that the activation of the motor-related brain areas, including the premotor areas (PMAs), the supplementary motor area (SMA), primary motor cortex (M1), the inferior parietal lobe (IPL), and the basal ganglia (BG), was altered, while behavioral performance, such as the finger-tapping frequency and accuracy rate, could be improved by training (Lafleur et al., 2002; Lacourse et al., 2005; Nyberg et al., 2006; Olsson et al., 2008; Zhang et al., 2011). Further studies based on GCM indicated that these brain areas also interacted with each other. Chen et al. explored the interactions between the SMA and other brain regions during a motor imagery task and found forward and backward interactions between the SMA and three regions: the bilateral dorsal PMA (dPMA), the contralateral primary and secondary somatosensory cortex (S1), and M1. They proved that the interaction of the SMA with other regions was closely related to brain lateralization of left- or right-hand imagery (Chen et al., 2009). Gao et al. investigated the directional influence among overlapped core regions recruited by motor execution and motor imagery tasks. The in-out degrees of the Granger direction at each ROI suggested that the contralateral dPMA, the IPL, and the superior parietal lobe (SPL) are causal sources of motor execution and motor imagery tasks, thus highlighting the dominant function of these regions (Gao et al., 2011). However, these studies regarding the causal relationship among the motor-related cortex primarily concentrated on motor imagery tasks or motor execution training; studies involving motor imagery training were very limited.

Based on the investigations described above, the current study attempts to explore the causal interaction of the target region with other motor-related regions during motor imagery training via rtfMRI. According to our previous work on off-line motor imagery training (Zhang et al., 2011), activities in the ipsilateral dPMA were highly correlated with motor performance, which suggested the dPMA was crucial to behavioral outputs of the motor execution task, and the improved motor performance relied more heavily on the functions of the dPMA. We chose the target region as the ipsilateral (right) dPMA accordingly. GCM was used to calculate the causal interaction between the right dPMA and the corresponding motor-related regions that were continually activated as the rtfMRI training progressed. We hypothesized that the activation of the right dPMA and other motor-related areas could be altered by rtfMRI training and that the directional interactions to and from the right dPMA also could be altered by such training.

## Materials and methods

### Ethics statement and subjects

The experiment was approved by the Institutional Review Board of the State Key Laboratory of Cognitive Neuroscience and Learning at Beijing Normal University. All subjects signed an informed consent form before participating in the scanning. Twelve healthy participants were assigned to the experimental group (mean age 23 ± 2.14 years, six males and six females). The other twelve participants (mean age 23 ± 1.7 years, eight males and four females) constituted the control group. All subjects had normal neurological examinations and were right-handed according to the Edinburgh Handedness Inventory (Oldfield, 1971). In addition, subjects experienced with typewriters and those with any training in musical instruments were excluded.

### Experimental procedure

The experimental paradigm was referenced from our previous study (Zhang et al., 2011) except that the repeated behavior training was replaced with rtfMRI feedback training. Outside of the scanner, all the participants were instructed that from their index to little finger, each of the four fingers of their right hand represented a single digit number: one, two, three, and four. Next, they were instructed to tap their right index finger with a metronome at 4 Hz to learn the rhythm required in the following scan session, after which they tapped the sequence 1-2-3-4 at 4 Hz for 30 s. Then, they tapped the set sequence 4-2-3-1-3-4-2 at 4 Hz for 30 s, and imagined tapping the set sequence at 4 Hz for another 30 s.

The experimental procedure in the scanner consisted of a pre-test, the rtfMRI training, and a post-test. In the pre-test, to anatomically delineate the individual target region (the right dPMA) for each participant, a functional localization task was performed, involving finger tapping of right hand (the sequence was 4-2-3-1-3-4-2 at a self-paced rate of 4 Hz). Blocks of 30-s of rest alternated with 30-s blocks of the finger tapping task, and the pre-test lasted 270 s. The sequence tapping was performed using a four-button response pad to record the behavioral data.

**Figure 1:**
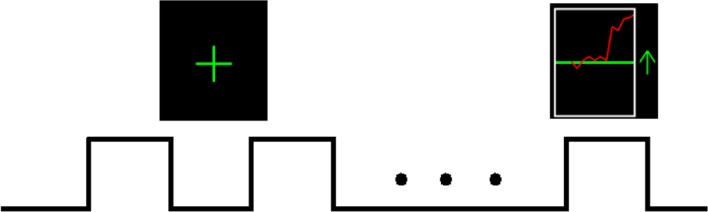
**Outline of one rtfMRI training session.** Each session lasted 450 s, during which time 30-s blocks of rest alternated with 30-s blocks of motor imagery with feedback, for a total of seven task blocks and eight rest blocks. During the rest blocks, green “+” sign was presented on the screen; and during the task blocks, the green up arrow appeared on the screen along with the continually updated red curve.

The rtfMRI training consisted of four sessions (TrainA, TrainB, TrainC, and TrainD). Each session included eight 30-s rest blocks interleaved with seven 30-s motor imagery block with feedback, and the feedback signal was presented to the subject as shown in Figure 1. When the green up arrow appeared on the screen, subjects were guided to imagine tapping 4-2-3-1-3-4-2 with their right fingers from a motor perspective at the pace of which they had just learned outside the scanner. The red curve was updated once per repetition time (TR) on the screen, which represented the activity of the target region. Subjects were instructed to maintain this value at the maximum possible level. The motor imagery strategy should be kinesthetic imagery of the movement of their right fingers and keep the sequence as 4-2-3-1-3-4-2, such as imagining playing the piano, pressing a thumbtack, or snapping one's fingers in the specified sequence. The red line feedback was postponed by 6 s to compensate for the inherent delay of the blood oxygenation level-dependent (BOLD) signal response. When the green “+” sign was displayed on the screen, subjects were guided to immediately stop their motor imagery training, keep themselves at a state of absolute rest, and try to think of nothing, especially thoughts associated with the task. In addition, when subjects were asked to imagine finger tapping, it was forbidden to control the feedback signal with actual movements, such as eye blinking, swallowing, or other small movements of the body. The subjects in the control group received the same experiment procedure and instructions except that they were supplied with a sham feedback signal taken from the experimental group.

Following the last training session, all of the participants were tested again in the scanner. The procedure and instructions of the post-test were identical to the pre-test. After the scanning, each subject was asked to complete a questionnaire to record the detailed strategies they used, the tapping pace, and any difficulty they encountered.

### On-line fMRI data acquisition and analysis

Brain images were acquired using a SIEMENS 3-T scanner at the MRI Center of Beijing Normal University. For each participant, T2^*^-weighted functional images were collected with the following parameters: *TR* = 2000 ms; echo time (*TE*) = 40 ms; slice = 32; matrix size = 64 × 64; slice thickness = 4 mm; inter-slice gap = 0.8 mm; flip angle (*FA*) = 90°; field of view (FOV) = 240 × 240 mm.

On-line data analysis was conducted using Turbo Brain Voyager software (Brain Innovation, Maastricht, Netherlands), including preprocessing and statistical analysis. Data preprocessing included 3D motion correction, drift removal, and spatial smoothing [full width at half maximum (FWHM) = 8 mm]. The statistical analysis was based on an incremental General Linear Model (GLM). The activation map with the significance *p* < 0.001 was updated once per TR and was presented to the experimenter for reference.

To cancel out global changes of BOLD, the background ROI was defined as a task-unrelated area in one slice away from the target ROI. The feedback signals presented to the subject were calculated as the difference between the mean BOLD value in the target ROI and the mean BOLD value in the background ROI using the following equation, which was updated once per TR:

(1) $${\left({\text{BOLD}}_{\text{training}}-{\text{BOLD}}_{\text{rest}}\right)}_{\text{targetROI}}\;-{\left({\text{BOLD}}_{\text{training}}-{\text{BOLD}}_{\text{rest}}\right)}_{\text{backgroundROI}}$$

### Off-line data analysis and time series extraction

Statistical parametric mapping 8 (SPM8) (http://www.fil.ion.ucl.ac.uk/spm/) was applied to process the fMRI data of the rtfMRI training sessions, including slice-timing, realignment, spatial normalization, smoothing, and GLM analysis for each subject. Group analysis was performed for each session in series (TrainA, TrainB, TrainC, and TrainD), and the brain areas with constant activation across sessions were selected as the ROIs [*p* < 0.05, cluster size >41, FDR (false discovery rate) correction]. For each ROI, the group spherical template was constructed using the spatial coordinates of maximum activation in the group activation map as the center with a 15 mm radius, while the individual spherical template was constructed using the spatial coordinates of maximum activation in each individual activation map as the center with a 6 mm radius. In each ROI, the BOLD time series from the normalized functional images before spatial smoothing was extracted from each voxel, located in the overlay areas of these two templates, within which activation intensity reached a specified value (*t* > 2.33).

### Granger causality analysis

Let *X* = [*x*(*n*)] and *Y* = [*y*(*n*)] be two BOLD time series, and if *y*(*n*) can be predicted more precisely according to the past information of both *x*(*n*) and *y*(*n*) than merely according to the past information of *y*(*n*) itself, *x*(*n*) is called the Granger causality of *y*(*n*). In the univariate case, *x*(*n*) and *y*(*n*) can be described by the autoregressive (AR) model as follows:

(2) $$\{\begin{array}{l} x(n)=\overset{p}{\underset{k\;=\;1}{\sum }}a_{1k}x(n-k)+u_{1}(n)\\ y(n)=\sum_{k\;=\;1}^{p}b_{1k}y(n-k)+v_{1}(n) \end{array}$$

Here, *u*_1_(*n*) and *v*_1_(*n*) are the prediction errors, and their variances σ_*X*|*X*^−^_ and σ_*Y*|*Y*^−^_ describe the accuracy of the prediction. In the bivariate case, the AR model is defined as follows:

(3) $$\{\begin{array}{l} x(n)=\sum_{k\;=\;1}^{p}a_{2k}x(n-k)+\sum_{k\;=\;1}^{p}c_{2k}y(n-k)+u_{2}(n)\\ y(n)=\sum_{k\;=\;1}^{p}b_{2k}y(n-k)+\sum_{k\;=\;1}^{p}d_{2k}x(n-k)+v_{2}(n) \end{array}$$

Similarly, *u*_2_(*n*) and *v*_2_(*n*) are the prediction errors, and the variances of *u*_2_(*n*) and *v*_2_(*n*) are σ_*X*|*X*^−^_,_*Y*^−^_ and σ_*Y*|*Y*^−^_,_*X*^−^_, respectively. The parameters of the AR model are estimated by the ordinary least squares (OLS) algorithm. The measurements of the Granger causality of *Y* to *X* and *X* to *Y* are defined as below (Geweke, 1982):

(4) $$F_{Y\;\to X}=\ln\frac{\sigma_{X|X^{-}}}{\sigma_{X|X^{-},Y^{-}}}$$

(5) $$F_{X\;\to Y}=\ln\frac{\sigma_{Y|Y^{-}}}{\sigma_{Y|Y^{-},X^{-}}}$$

The order of the AR model in equations (2) and (3) can be determined by minimizing the Akaike information criterion (AIC), which is defined as:

(6) $$AIC(p)=N\ln\left\{\det\left[\sum \left(p\right)\right]\right\}+2pM^{2}$$

Here, *M* is the dimension of AR model, and in this study, *M* is 2; *N* is the number of the time points, and ∑(*p*) is the variance of the prediction of the *p*th-order model. For two given BOLD time series, the value of *AIC*(*p*) decreases when the order *p* increases. When *AIC*(*p*) reaches the minimum, the value of *p* achieves the optimal order. In this paper, the order of the AR model was chosen to be 5 based on AIC.

Before calculating the Granger causality between two ROIs, the BOLD time series must be preprocessed. First, for each voxel, global effects were removed using the global mean scaling function in SPM8 to stabilize the time series at a session level. Second, for each training session, the data across 7 task blocks was averaged to obtain the task-level mean value. This task-level mean value was subtracted from the BOLD time series in each task block. Third, for each task block, the temporal mean value was computed and removed to meet the zero mean requirement assumed by the AR model (Ding et al., 2000, 2006). After preprocessing, for each given pair of ROIs, the Granger causality was calculated for all pair-wise combinations of voxels and then averaged (Wen et al., 2012, 2013).

In order to analyze the changes resulting from rtfMRI training, we focused only on the interaction differences across the sessions using a Wilcoxon test. The possible confounding effect of the inter-regional variability of hemodynamic response was thus avoided since it is unlikely that the inter-ROI hemodynamic differences can change across sessions (Roebroeck et al., 2005; Seth, 2010; Tana et al., 2012; Bianchi et al., 2013). Our focusing only on the presence of session-modulated casual influence allows us also to avoid a comparison with surrogate data. Indeed, the statistical analysis of Granger index without surrogate data can also provide statistically reliable results if the causality differences between sessions were evaluated rather than the causality induced by the session itself (Seth et al., 2013).

## Results

### Behavioral performance

**Figure 2:**
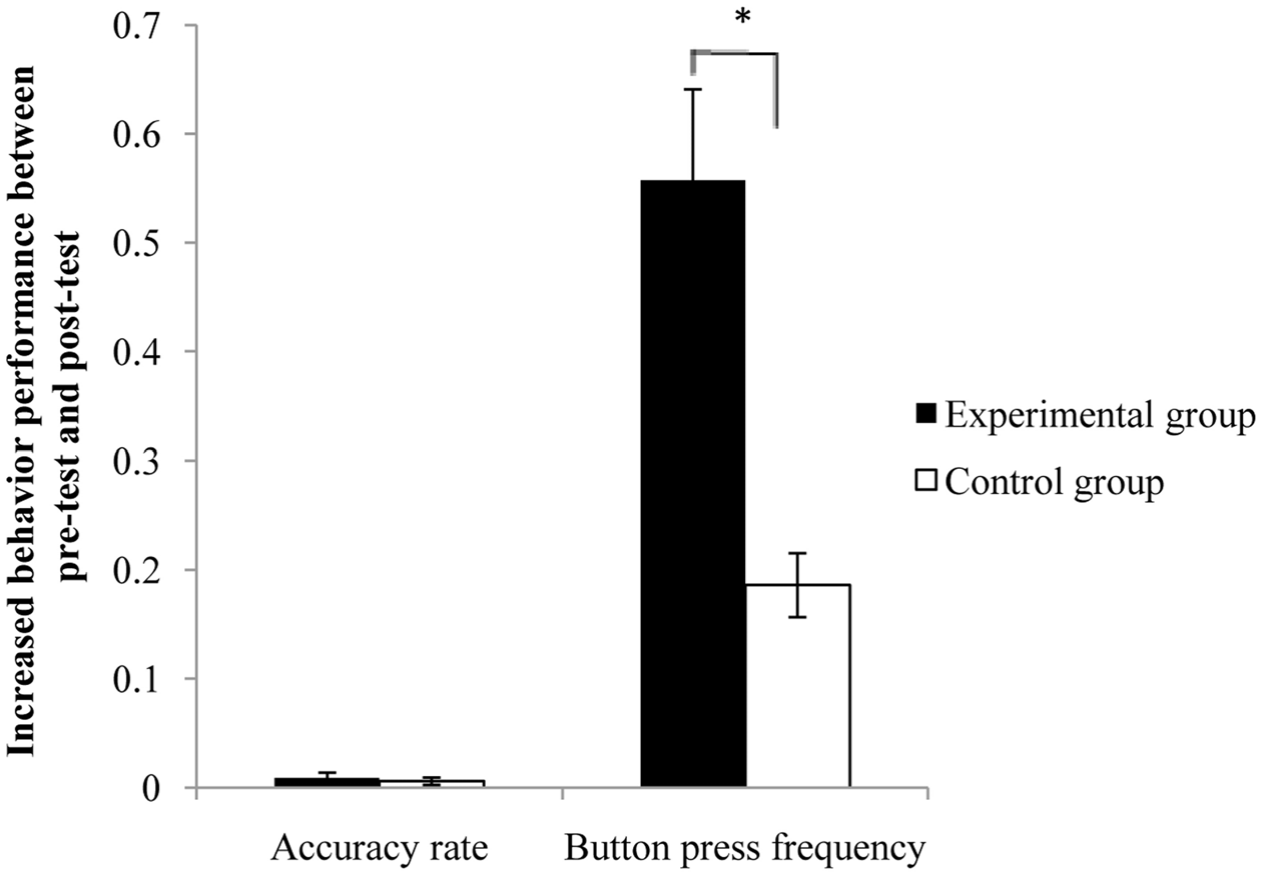
**Behavioral changes between the pre- and post-test for the two groups.** The increase of button press frequency from pre-test to post-test was significantly greater in the experimental group than that in the control group. Error bar means the standard error. *p < 0.05.

The completion of button pressing was recorded for the motor execution task inside the MRI scanner during the pre-test and post-test sessions. The mean button press frequency and accuracy rate were calculated for each test. Comparison of the button press frequency and accuracy rate between pre-test and post-test conditions in each group and the comparison of changes between the two groups were displayed in Table 1 using a *t*-test. Although the behavioral performance was increased in both groups after the rtfMRI training, between-group comparison of behavioral changes indicated that the increase of button press frequency in the experimental group was significantly greater than that in the control group (Figure 2).

**Table 1:** **Behavioral performance in the experimental group and the control group before and after the rtfMRI training**.

	**Pre-test Mean (SE)**	**Post-test Mean (SE)**	**Post vs. pre**	**Group difference^a^**
**BUTTON PRESS FREQUENCY**				
Experimental group	2.258 (0.131)	2.815 (0.181)	*p* < 0.001	*p* < 0.001
Control group	1.888 (0.126)	2.074 (0.115)	*p* < 0.001	
**ACCURACY RATE**				
Experimental group	0.956 (0.009)	0.964 (0.006)	*p* < 0.05	*p* = 0.32
Control group	0.957 (0.008)	0.963 (0.005)	*p* < 0.05	

SE, standard error.

a Comparison of behavioral changes from pre-test to post-test in the experimental group with that in the control group.

### Brain activation and definition of ROIs

**Figure 3:**
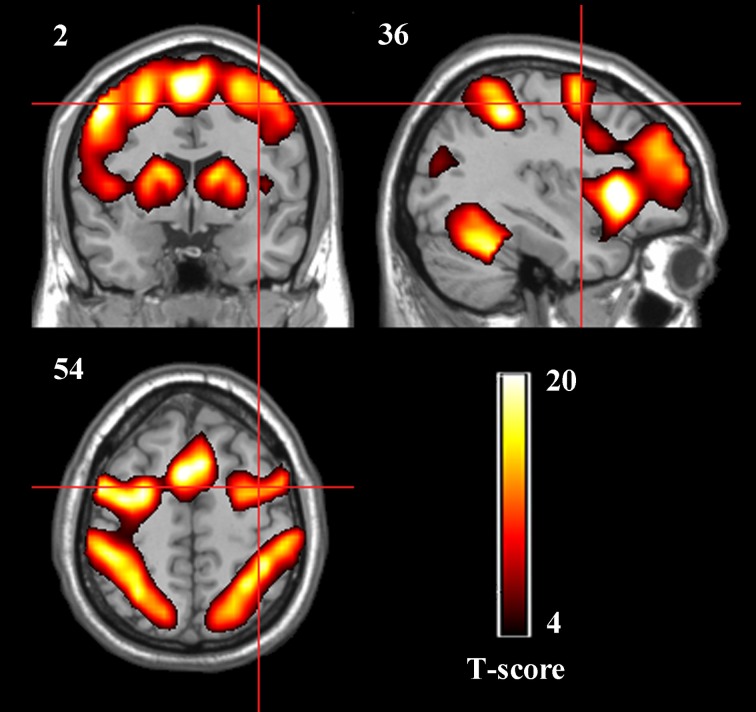
**Group activation maps of the two groups together during the rtfMRI imagery training (*p* < 0.05, cluster size > 41, FDR correction).** The peak value in the right dPMA was located at the MNI coordinates *x* = 39, *y* = −1, *z* = 42.

The spatial activation patterns across the four rtfMRI training sessions in the two groups displayed stability and consistency, including the bilateral dPMA, SMA, contralateral M1, SPL, BG, and cerebellum. Whole-brain analysis showed that there was no significant difference between TrainD and TrainA both in the experiment group and in the control group, and only significant difference between TrainC and TrainD in the experiment group, which contained increased activation in the target region, as well as part of the left dPMA and SMA (paired *t*-test, *p*<0.001, cluster>10, uncorrected) in TrainD. According to these results, nine ROIs were selected: left dPMA, right dPMA, SMA, left M1, cerebellum, left SPL, right SPL, left BG and right BG (Figure 3).

### Signal changes in ROIs during rtfMRI training

The percent signal change was calculated as the differential of mean BOLD in the task block with that in the rest block divided by the mean BOLD of the rest block. Two-Way repeated-measures ANOVA with main factors of session (four sessions; within-participants) was performed using SPSS 17.0 (SPSS Inc., Chicago, IL, USA) to examine the differences of percent signal changes between sessions at each ROI. There was a significant main effect of session at the SMA [*F*_(3, 33)_ = 4.219, *p* < 0.05] and left M1 [*F*_(3, 33)_ = 4.885, *p* < 0.05] in the experiment group, and at the right SPL [*F*_(2.03, 22.09)_ = 4.57, *p* < 0.05) in the control group. The percent signal changes of SMA and left M1 in the experiment group increased in a linear trend (linear regression analysis, SMA: *R*^2^ = 0.969, *p* < 0.05; left M1: *R*^2^ = 0.643, *p* = 0.198), and the percent signal changes of right SPL in the control group decreased in a linear trend (*R*^2^ = 0.6979, *p* = 0.165).

**Figure 4:**
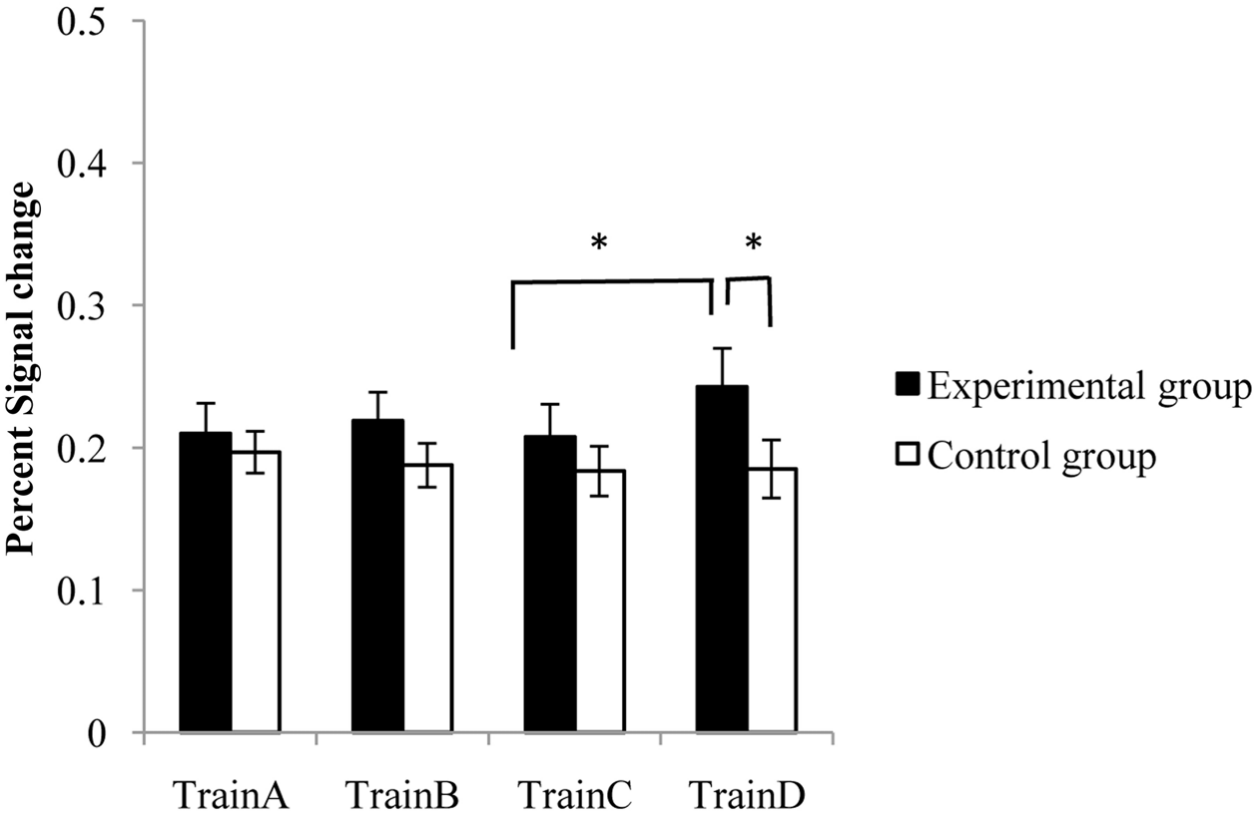
**The percent signal changes of the target ROI in the experimental group and the control group during rtfMRI training sessions.** Error bar means the standard error. ^*^p < 0.05.

No significant effect of session was observed in the target ROI of both the experiment group and control group. A pair-wise comparison analysis suggested that compared with session TrainC, significant increases of activation in the target ROI were observed in session TrainD [paired *t*-test, *t*_(11)_ = 3.037, *p* < 0.05] in the experiment group. A marginal significant difference of activation was also observed between TrainB and TrainD [*t*_(11)_ = 1.59, *p* = 0.0706], but there was no significant difference between TrainA and TrainD [*t*_(11)_ = 1.095, *p* = 0.148]. No significant differences were detected between any rtfMRI training session pairs in the control group, but the difference between the two groups in TrainD was significant (two sample *t*-test, *p* < 0.05) (Figure 4).

### Granger causal interaction of the target ROI

**Figure 5:**
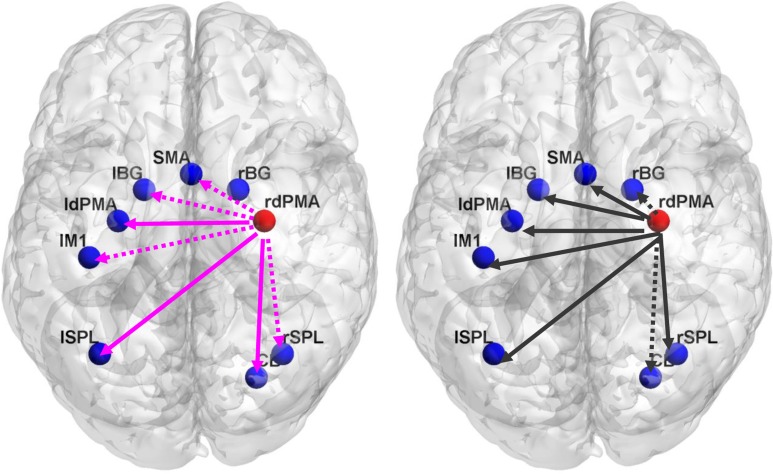
**The difference of Granger causality index between TrainD and TrainA in the experiment group (left) and the control group (right).** The pink color line represents the increased Granger index from TrainA to TrainD, and the black color line represents the decreased index from TrainA to TrainD. The solid line represents the significance level *p* < 0.05, whereas the dotted line represents the marginal significance level 0.05 < p < 0.1. ldPMA, left dPMA; rdPMA, right dPMA; lM1, left M1; lSPL, left SPL; rSPL, right SPL; lBG, left BG; rBG, right BG.

For each subject, bidirectional Granger causality indices between the target ROI and the other eight ROIs were calculated for each training session in the two groups. Wilcoxon test of Granger causality indices between TrainA and TrainD was performed to assess alterations in the causal interactions by rtfMRI training. The analysis revealed significant changes in the indices from the target ROI to the other ROIs in TrainD for both two groups (Figure 5). In contrast, the indices from the other ROIs to the target ROI did not exhibit any consistent trend. Moreover, the linear regression analysis of the Granger index across the training sessions showed a progressively increase of unidirectional interaction from the target region to all other ROIs in the experiment group, but a progressively decrease of unidirectional interaction also from the target region to all other ROIs in the control group, although the linear trend was not significant.

## Discussion

The present study elucidated the dominant role of the target region in rtfMRI training from the causal interactions perspective. As predicted, we found that (1) the activation of the motor regions and behavioral performance of motor execution were altered by rtfMRI training, and (2) the causal interaction was also changed by rtfMRI training with stronger influences from the right dPMA to other related regions. In essence, these results demonstrated a significant increase in the unidirectional interaction from the target region.

After rtfMRI training, button press accuracy and frequency improved significantly (Figure 2), implying that the increase of tapping rate was not at the cost of increased rate of error. This result was consistent with previous studies (Driskell et al., 1994; Ranganathan et al., 2004; Hotermans et al., 2006). Although the obvious within-group enhancements were detected in the two groups, the improvement of performance in button press frequency was significantly greater in the experimental group than that in the control group. This improvement difference of behavior performance between the two groups indicated that motor imagery training with true neurofeedback is more effective in improving the motor behavior than that with sham neurofeedback. The behavioral performance results suggested that motor function relied more heavily on the functions of the right dPMA, such as motor planning and motor organization (Nakayama et al., 2008; Zhang et al., 2011). On-line rtfMRI training can achieve the similar improvement of behavioral performance as off-line training, which provides a novel method of motor imagery training.

During rtfMRI training, the congruence in functional neuroanatomy primarily converged on the SMA, dPMA, left M1, SPL and cerebellum, which was also reported in other studies (Hanakawa et al., 2002; Hugdahl, 2009). The analysis of ROIs revealed that the significant main effect of session appeared at the SMA and the contralateral M1 in the experiment group, and the ipsilateral SPL in the control group. Although no significant main effect of session was found in the target region in both groups, a fluctuating rising trend of the signal changes in the right dPMA was observed in the experiment group but not in the control group, in agreement with previous reports. For example, in one study by Weiskopf et al., subjects were trained to regulate the BOLD signal difference between the SMA and the parahippocampal place area (PPA), and the signal increased in a fluctuating manner during nine tasks (Weiskopf et al., 2004). Another rtfMRI study of M1 by Yoo et al. also indicated that the signal change of the target region appeared to trend upwards in a fluctuating manner as the training progressed (Yoo et al., 2008). Moreover, the signal change of the right dPMA in TrainD in the experiment group was significantly higher than in TrainC, and was also significantly higher than that in the control group. According to the hypothesis of Karni and Toni (Karni et al., 1995; Toni et al., 1998), the observed fluctuating effect may be due to two parallel mechanisms: an enhancement mechanism that caused the initial increase and a repetition suppression mechanism that was engaged by continuous feedback training. However, the obviously increased signal changes of the experimental group in train D (Figure 4) indicated that participants receiving true feedback have gradually learned the skill to control the activation of right dPMA. The results proved our hypothesis that regulating on the right dPMA by rtfMRI training could lead to the alteration of activation in the other motor-related regions. Previous studies have highlighted the critical roles of motor cortical areas such as SMA, M1 and dPMA in motor sequence learning, including motor preparation, planning, processing, and output of the motor task (Shibasaki et al., 1993; Rao et al., 1998; Lotze et al., 1999). The significant improvement of tapping speed aligned with the activation changes of these cortical regions, which were dependent on the regulation of the target region, suggesting the tight interactions between the target region and other function-related regions.

Previous studies have indicated that the activity of motor-related regions, such as the SMA, M1, dPMA, SPL, BG and cerebellum, could be altered through motor imagery training, and causal interactions existed between these brain areas (Chen et al., 2009; Ma et al., 2010; Gao et al., 2011). Our study not only demonstrated changes of the activation in these regions by rtfMRI training but also revealed the altered directional interaction between them, especially from the target region. As shown in Figure 5, there was a opposite trend of the changes in causal interactions from TrainA to TrainD in the two groups, and the significant changes of interaction in the two groups both appeared in the direction from the target region to other regions. These significant changes might be attributed to the dominant role of the dPMA over other regions involved in motor sequence learning. First, the SMA and dPMA both function in movement planning (Shibasaki et al., 1993; Nakayama et al., 2008). Studies have shown that activation of the SMA increased with the familiarity of motor task (Hikosaka et al., 1996; Grafton et al., 2002), especially in the motor sequence learning task. These studies illustrated that the SMA played a critical role which were rehearsed from memory and fitted into a precise timing plan (van Mier et al., 1999). Thus, the notable decrease interaction from the dPMA to the SMA in the control group by the training may have reflected the runaway tendency to link in sequence movement planning. Second, the SPL plays the important roles of receiving and analyzing somatosensory information in the early stage (Binkofski et al., 2000; Buccino et al., 2001) and memory of motor skills in the later stage of learning (Hazeltine et al., 1997; Ghilardi et al., 2000). Imagery strategy in our study was instructed to be performed from a motor perspective not a visual perspective. The opposite changes of the Granger index from the right dPMA to the SPL in the two groups may state that the true feedback causes an enhancement of the transformation and integration of sensorimotor information. Third, it is well accepted and cited that M1 is a brain region tightly linked to motor execution and motor output (Shibasaki et al., 1993; Rao et al., 1998; Lotze et al., 1999). The decreased influence of the right dPMA on the M1 might induce the significantly lesser changes of tapping movement in the control group than that in the experiment group after training. Fourth, both the cerebellum and BG are related to performing a movement, in which the cerebellum is mainly involved in optimizing movements using sensory feedback, and the BG is mainly concerned with the appropriate movement selection (Jueptner and Weiller, 1998; Sakai et al., 2000). The different feedback stimuli in the two groups might confound the movement selection and adjustment, mainly reflected in that the increased influence of the right dPMA on the cerebellum in the experiment group and the decreased influence of the right dPMA on the BG in the control group. The post-training questionnaire also showed that most participants in the control group couldn't find the effective strategy to realize the successful regulation by the feedback.

It should be noted that the trend of unidirectional interaction from the target region to other motor related regions is similar with the trend of signal changes at the target region in a gradually rising manner as the training progressed in the experiment group. Together with the changes in regional activation, the increased interaction from the right dPMA to other regions might reflect the process by which subjects learned to control the activity of the target region. Functional imaging studies showed that motor skill learning has different stages including fast learning in the early stage to establish a basic relationship between cortical activities and slow learning in the later stage of a consolidated and sustainable development process (Karni et al., 1995, 1998). In the early stage of training, all the other motor related regions exhibited enhanced interaction with the target region to support for the self-control of the activation in the target region. The gradually increased interaction in the experiment group suggested that the activities of other motor related regions became much more dependent on the regulation of the target region by well-rehearsed training. In the later stage of training, the variance of the changes in directional interaction might be due to the repetition effect (Kandel et al., 1991; Fuster, 1995) and the subjects' expectation, leading to more pronounced changes in the last training session.

Consistent with existing studies of motor imagery training, our study based on rtfMRI training revealed the causal interaction between the target region and other motor-related regions. The changes in the Granger causal index following the alterations in regional activation and behavioral performance suggest that information exchange in motor planning and sequence control gradually increased during rtfMRI. Our findings extended the previous studies of the points of interaction, implying that the dPMA played the dominant role in motor skill learning.

### Conflict of interest statement

The authors declare that the research was conducted in the absence of any commercial or financial relationships that could be construed as a potential conflict of interest.
